# Determination of Programmable Shunt Setting Using CT: Feasibility Study

**DOI:** 10.7759/cureus.19818

**Published:** 2021-11-22

**Authors:** Einat Slonimsky, Brad Zacharia, Alex Mamourian

**Affiliations:** 1 Diagnostic Radiology, Penn State Health Milton S. Hershey Medical Center, Hershey, USA; 2 Neurosurgery, Brain Surgery, Penn State Health Milton S. Hershey Medical Center, Hershey, USA; 3 Radiology, Penn State Health Milton S. Hershey Medical Center, Hershey, USA

**Keywords:** programmable shunts, metal artifacts, computed tomography, hydrocephalus, dual energy ct

## Abstract

Background: Programmable shunts can be adjusted to optimize CSF diversion in patients with hydrocephalus without the need for re-operation. Currently, all shunts incorporate radiopaque markers so that their setting can be determined on skull X-ray images. The purpose of this study was to evaluate whether the shunt setting could also be determined ex vivo and in vivo using the data from a standard head CT scan since one is nearly always obtained when patients with VP shunts present with new symptoms that could be due to shunt malfunction.

Materials and Methods: Four commonly used programmable shunts were attached to a dried skull and scanned using a variety of CT techniques. The shunts imaged were the Certas^TM^ Plus (Codman, Raynham, Massachusetts), Polaris^®^ (Sophysa, Orsay, France), proGAV 2.0^®^ (Braun, Bethlehem, Pennsylvania), and Hakim^®^ (Codman, Raynham, Massachusetts). Each shunt was scanned at two different valve settings using multiple CT techniques: CTDI_vol_ 75, 140kVp, 330mAs, CTDI_vol_60, 120kVp 390mAs, CTDI_vol_40, 80kVp with 430mAs, 140kVp with 215mAs. Image reconstruction with and without CT metal suppression software was used for all scans, and the data was reconstructed into volume-rendered images. We enlisted ten observers to review the volume-rendered images only. After a short set of training slides viewed by all observers, they were asked to predict the shunt setting for each valve along with their level of confidence. One clinical case of a patient with a programmable valve was evaluated on a CT scan.

Results: Using the volume-rendered images only, the two shunt settings of the Polaris shunt were correctly predicted by all the observers and in nine of 10 settings for the Certas^TM^ Plus valve. For the Hakim^®^ shunt and the proGAV 2.0^®^ shunt, setting prediction accuracy was 0% and 10%, respectively. In one clinical case, the programmable valve setting could be determined from the CT scan data.

Conclusion: The valve setting of at least two currently available programmable shunts can be determined using volume-rendered images generated from CT data. Reconstructions using metal suppression software were rated as superior and may be necessary for some valve designs.

## Introduction

Hydrocephalus is a common brain disorder that can result in damage to the brain parenchyma and its function [1]. It affects between 1% and 2% of the population [2]. Symptomatic patients are often treated with ventriculoperitoneal shunt (VPS) placement, but these have up to a 30% failure rate within the first year [3], so frequent follow-up, including radiographic evaluation, is routine. Currently, VPS may have either a fixed or programmable valve at the junction of the ventricular and distal catheter. Devices using programmable valves are more expensive than those with a single fixed setting but allow for non-invasive alteration of the valve setting to titrate hydrocephalus management. Several studies have demonstrated the benefit of using programmable valves since they can provide better neurological results in patients while remaining cost-effective when compared to non-programmable valves [4]. A recent metanalysis indicated that programmable valves can reduce the revision rate and the over- or under-drainage complication rate in patients under 18 years of age [5].

The programmable valve adjustment on all current devices is performed transcutaneously using a magnet placed on the skin overlying the valve. The valve setting can also be measured using a hand-held device, but these are valve specific. Plain skull radiographs are commonly used to determine valve settings since they allow the determination of the setting of any programmable valve without specialized equipment since reference images are provided online by the manufacturers and in the literature [6]. Since valve adjustment is performed using a hand-held magnet, the valve setting in some programmable valves can be inadvertently altered by a strong magnetic field, for example, during an MRI scan or exposure to powerful magnets used in toys and some electronic devices [3]. There is one report of a suicide attempt after a patient intentionally and successfully altered their valve with a handheld degaussing device [7]. While the latest designs of programmable devices incorporate mechanical interlocks that provide some protection from inadvertent valve changes, validation of settings after MRI is still recommended for all devices.

When a patient presents to the ER with symptoms suggesting shunt malfunction, routine imaging usually includes plain x-ray imaging of the head and neck to evaluate for shunt tubing continuity and a CT scan to evaluate the patient’s ventricular size. Radiographs are recommended to optimally demonstrate the programmable valve setting that requires orienting the direction of the X-ray beam perpendicular to the valve rather than using standard skull views. We are not aware of any efforts by manufacturers to incorporate into their valve designs material choices that would allow their valve settings to be determined from CT alone, but with newer valve designs and the wide availability of CT metal artifact suppression techniques, we hypothesized that shunt valve setting may be readily determined via CT alone. 

Historically, metal-related artifacts that include beam hardening and photon starvation severely limit the use of diagnostic CT for imaging any implanted metallic device [8,9]. Artifacts created by brain implants such as aneurysm clips and endovascular coils may obscure both implants as well as nearby brain tissue [10] because there is either insufficient or erroneous X-ray attenuation data that degrades the quality of image reconstruction. While reports have indicated that titanium aneurysm clips can be made more visible by modifying the CT technique used for image data acquisition [11] recently, many clinical CT scanners use sophisticated image reconstruction software that provides effective metal artifact suppression [12,13]. We hypothesized that CT imaging of a programmable valve, facilitated by a combination of modified CT technique and metal suppression reconstruction software, would allow the determination of its setting. This option may be of benefit, particularly when then the ventricular size has changed from prior scans, and especially valuable when plain films are unavailable or suboptimal. We utilized a phantom for scanning four commonly used programmable shunts to evaluate the feasibility of this approach and provide an in vivo example.

## Materials and methods

Phantom model and study design

Major shunt manufacturers currently offering programable shunts were contacted and asked to provide shunts and their adjustment hardware for this study. We were able to obtain four programmable shunt valves: CertasTM Plus (Codman, Raynham, Massachusetts), Polaris® (Sophysa, Orsay, France), proGAV 2.0® (Braun, Bethlehem, Pennsylvania), and Hakim® (Codman, Raynham, Massachusetts). The tools and instructions for their adjustment and radiographic appearance of each of these programmable shunt valves are readily available [6]. 

**Figure 1:**
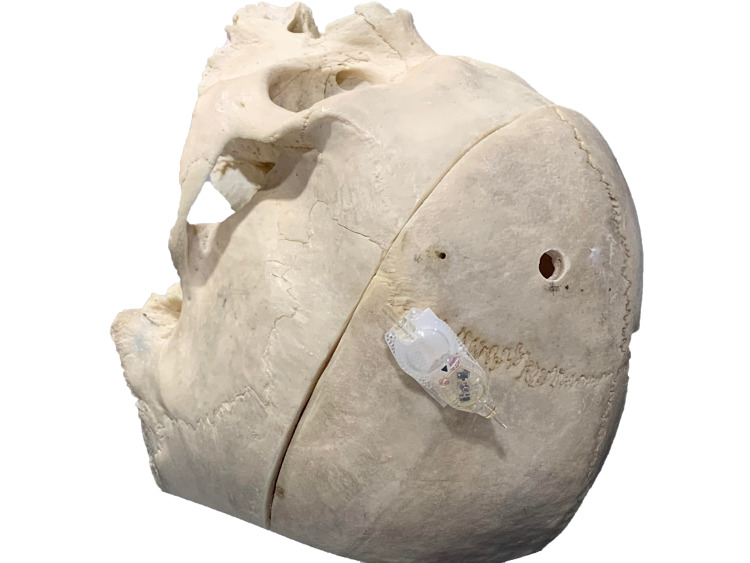
The phantom, scanned with two different shunts taped to the left and right parietal bones of the skull A dried human skull was used as the phantom and scanned with CertasTM Plus shunt taped to the parietal bone to replicate the beam hardening that is expected to occur in patients from the skull immediately adjacent to the shunt valve.

A dried human skull was used for our phantom in order to partially replicate the beam hardening that contributes image artifacts on CT reconstructions. The phantom was scanned in the usual anatomic position using a clinical scanner (Siemens-Erlangen 128 slices FLASH scanner, Germany) with the programmable shunts taped to the outer table of the skull phantom (Figure 1).

In the first part of the study, the phantom was scanned with two different shunts taped to the left and right parietal bones of the skull. The phantom was then scanned using three different scanner technique settings: (1) 140kVp that provided a relatively high dose (CTDIvol 75mGy), (2) our standard head CT scan technique using kVp 120, and (3) dual-energy CT technique (Table 1). 

**Table 1:** CT scan parameters for various shunts for both shunt settings

		Certas^TM^ Plus	Polaris^®^	proGAV 2.0 ^®^	Hakim^®^
Position 1	Scan 1	CTDI_vol_ 75, 140kVp, 330 mAs	CTDI_vol_ 75, 140kVp, 330 mAs	CTDI_vol_ 75, 140kVp, 330 mAs	CTDI_vol_ 75, 140kVp, 330 mAs
	Scan 2	CTDI_vol_ 60, 120 kVp, 390 mAs	CTDI_vol_ 60, 120 kVp, 390 mAs	CTDI_vol_ 60, 120 kVp, 390 mAs	CTDI_vol_ 60, 120 kVp, 390 mAs
	Scan 3	CTDI_vol_ 40 80 kVp 430 mAs 140 kVp 215 mAs	CTDI_vol_ 40 80 kVp 430 mAs 140 kVp 215 mAs	CTDI_vol_ 40 80 kVp 430 mAs 140 kVp 215 mAs	CTDI_vol_ 40 80 kVp 430 mAs 140 kVp 215 mAs
Position 2	Scan 4	CTDI_vol_ 75, 140 kVp, 330 mAs	CTDI_vol_ 75, 140 kVp, 330 mAs	CTDI_vol_ 75, 140 kVp, 330 mAs	CTDI_vol_ 75, 140 kVp, 330 mAs
	Scan 5	CTDI_vol_ 60, 120 kVp, 390 mAs	CTDI_vol_ 60, 120 kVp, 390 mAs	CTDI_vol_ 60, 120 kVp, 390 mAs	CTDI_vol_ 60, 120 kVp, 390 mAs
	Scan 6	CTDI_vol_ 40 80 kVp 430 mAs 140 kVp 215 mAs	CTDI_vol_ 40 80 kVp 430 mAs 140 kVp 215 mAs	CTDI_vol_ 40 80 kVp 430 mAs 140 kVp 215 mAs	CTDI_vol_ 40 80 kVp 430 mAs 140kVp V 215 mAs

Reconstructions with and without Siemens metal suppression iterative image reconstruction software (iMAR) was used for all the scans. This was repeated so that eventually, all four shunts were imaged in the same way.

In the second part of the phantom study, the setting of each of the shunts was changed and confirmed using the specific hand tools provided by the manufacturers. The shunts were then imaged utilizing the aforementioned technique.

CT imaging, data acquisition and post-processing

CT scan for each shunt in two different positions included the following parameters:

1. CTDIvol 75, 140kVp, 330 mAs

2. CTDIvol 60, 120 kVp 390 mAs

3. CTDIvol 40, 80 kVp with 430 mAs, 140 kVp with 215 mAs

The shunt settings are detailed in Table 2. 

**Table 2:** Shunt parameter setting for each programable shunt on both scans

	Certas^TM^ Plus	Polaris^®^	proGAV 2.0 ^®^	Hakim^®^
Side	Right	Left	Right	Left
Position 1	4	2	15	6
Position 2	7	4	170	70

**Figure 2:**
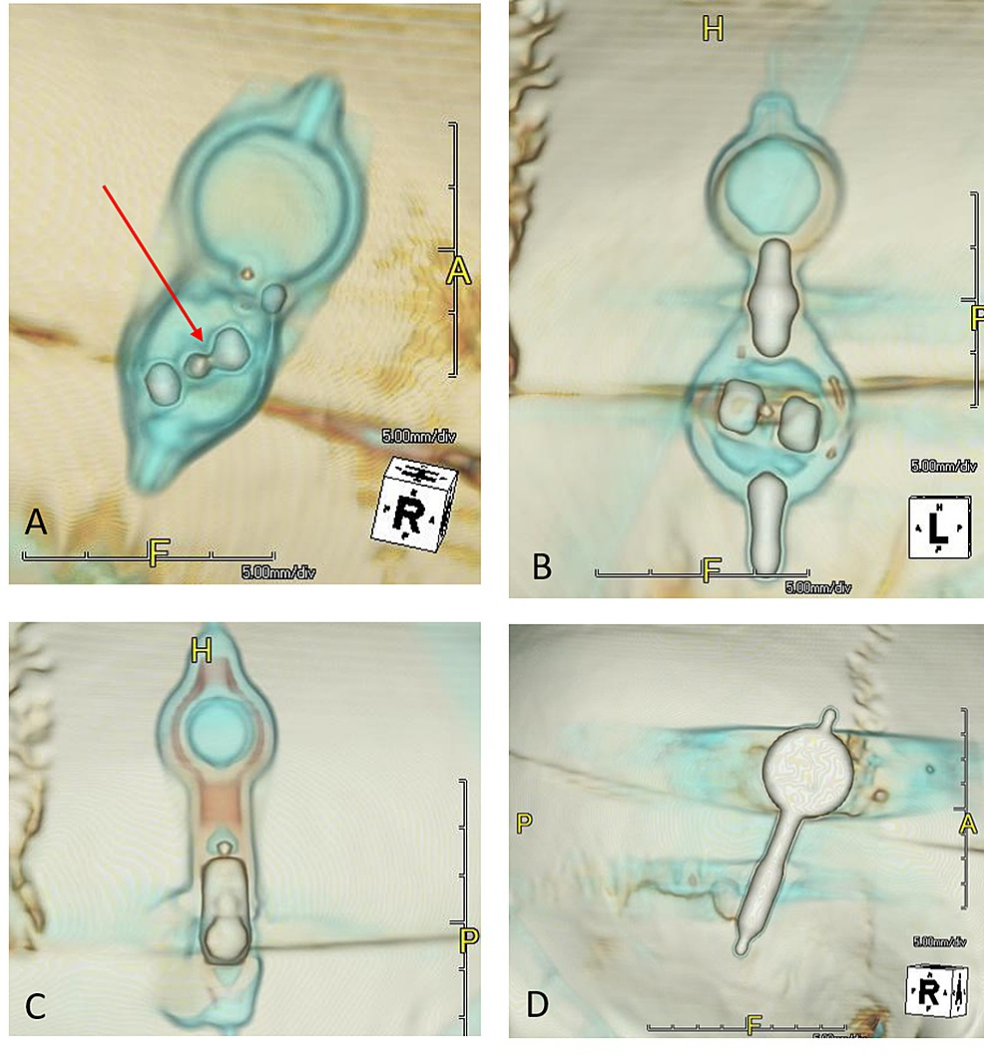
Volume reconstructions of the four shunts (A) CertasTM Plus (Codman, Raynham, Massachusetts).  (B) Polaris® (Sophysa, Orsay, France). (C) Hakim® (Codman, Raynham, Massachusetts). (D) proGAV 2.0® (Braun, Bethlehem, Pennsylvania). Note the difficulty in detection of detail within the valves on volume rendered images (C) and (D). While the two lobes on the Certas valve (A) and their angle relative to the reference indicator (long arrow) are evident, it is essential to recognize the slight difference in the appearance of the two lobes. A small tab is present on only one lobe, and this is indicated on the volume rendered by the short red arrow. Correct identification of each of the two lobes is critical for correct setting prediction. The two lobes of the Polaris valve (B), on the other hand, are identical and only a limited range of angles are available relative to the long axis of the valve. This arrangement is very likely why we found a high degree of accuracy in determining the valve setting among our 10 observers.

Post-processing was performed using Terarecon 3D software (Durham, NC). A standard 3D reconstruction template that was available from the software menu was used to create volume-rendered images of the valve as though viewing the valve from outside the skull and were saved as .jpg images for each device at each setting (Figure 2).

Questionnaire study design

To validate our observations, we created an education set that included a short set of volume-rendered images of the different valves so that the observers could learn what to look for when imaging programmable valves with CT. Ten radiology residents at different training levels were asked to evaluate the phantom CT-based volume rendered images. Following a short training set of images that indicate the key findings to look for (approximately five minutes), a quiz was presented to each that included 16 questions and eight images. Participants were asked to indicate the shunt settings of the four valves and to report their confidence with regard to that prediction using a scale of 1-5, with five indicating total confidence. Immediately after this test, the same subjects were asked to rate a set of volume-rendered images to select the three best quality volume-rendered images, among eight presented, that they perceived as providing the best demonstration of fine detail within only two of the valves, CertasTM Plus and Polaris®. The volume-rendered images in that last section of the questionnaire were created using data from the CT using 140kVp with and without iMAR, 120 kVp with and without iMAR, and dual-energy CT at both 80 kVp and 140 kVp with and without iMAR, for a total of eight images. The participants were not allowed to ask questions and did not receive any coaching during either part of their valve imaging evaluation.

In vivo scan

Data from a routine, clinically indicated, non-contrast head CT study of a patient with a programmable valve (CertasTM Plus) was used for this purpose. The patient was scanned with our standard imaging technique: kVp 120, mAs 243, FOV 240, CTDI vol 58. volume-rendered images were created using thin reconstructions (0.75mm) with and without iMAR. The shunt setting was predicted based on the volume-rendered images and compared to the data from the medical record.

Statistical analysis

Descriptive data were entered and analyzed with an electronic spreadsheet using the MS Excel software program (Microsoft Corporation, Redmond, WA). 

## Results

For the Polaris® shunt, the setting was read correctly by 10/10 (100%) of the participants at both shunt settings. Their average confident interval was 4.2 for both settings.

For the CertasTM Plus shunt, the setting was read correctly by 9/10 (90%) at one setting with a confidence of 4.0 and by 10/10 (100%) at the second setting with a confidence of 4.4.

For the Hakim® shunt, the setting was read correctly by 0/10 (0%) in the first setting with the confidence of 1.4 (range 1-5), and by 0/10 (0%) in the second setting with the confidence of 1.4.

For the proGAV 2.0® shunt, the setting was read correctly by 1/10 (10%) in the first setting with the confidence of 1.7 and by 0/10 (0%) in the second setting with a confidence of 1.6 (Table 3).

**Table 3:** Evaluation of shunt setting based on volume rendered images from phantom-CT scans *CI: confidence interval (0-5)

	Correct read of shunt setting	
	Setting 1 (CI*)	Setting 2 (CI*)
Polaris^®^shunt	10/10 (4.2)	10/10 (4.2)
Certas^TM^ Plus shunt	9/10 (4.0)	10/10 (4.4)
Hakim^®^ shunt	0/10 (1.4)	0/10 (1.4)
proGAV 2.0^®^ shunt	1/10 (1.7)	0/10 (1.6)

In the subjects, evaluation of volume-rendered reconstructions of two of the valves, the three best rated volume-rendered images of both the Polaris® valve and Certas Plus valve used CT data from scans acquired with 140kVp processed with iMAR reconstruction, non-contrast CT at 120kVp with iMAR reconstruction, and a dual energy non-contrast CT (140kVp) with iMAR.

**Figure 3:**
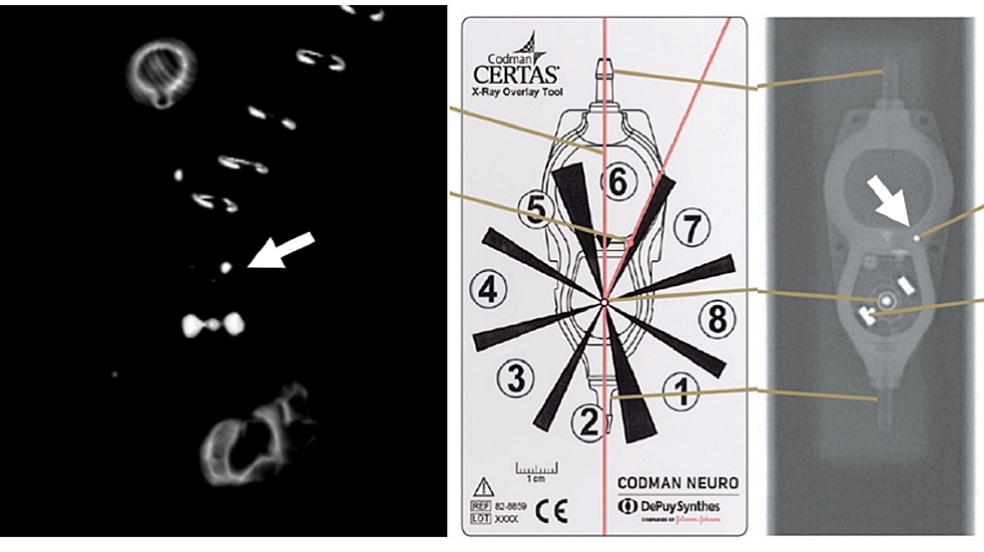
CertasTM Plus volume rendered image reconstruction from routine non-contrast head CT The shunt setting is predicted compared with the manufacturing guide and was correctly predicted as four. The lobes that come closest to the center marker is the one with the extra metal dot, assisting in reading the setting correctly. The setting is compared with the manufacture guidelines in relation to the radiopaque marker (arrow).

In our single included patient, the setting of the CertasTM Plus was correctly predicted using CT data as number four (Figure 3).

The setting was determined using volume-rendered reconstructions of the thin slices (0.75mm), with a minimal added benefit when the image reconstructions were processed using our metal suppression software (iMAR).

## Discussion

**Figure 4:**
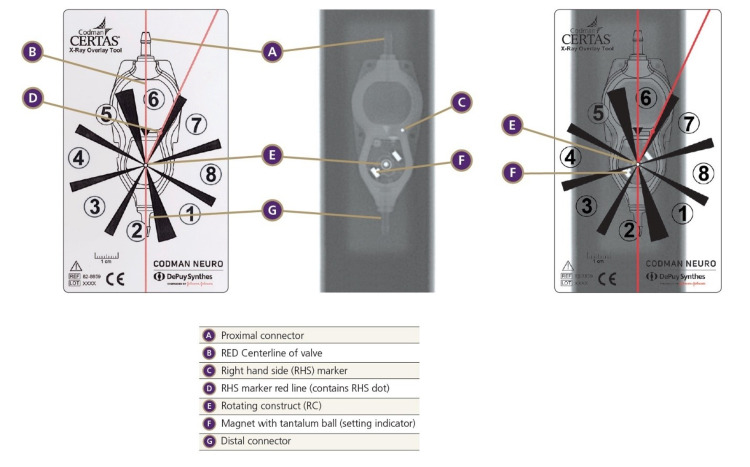
CertasTM Plus valve manufacture guide for interpretation of shunt setting on x-Ray

Using only 3D volume-rendered reformations of CT data, the participants were all successful in determining the correct shunt setting for the Polaris® at both settings. Their performance was nearly as good for the CertasTM Plus valve. None of the participants could determine either of the shunt settings for the Hakim® and proGAV 2.0® valves. We believe this is due to the metal casing of those valves, as well as the design of the internal structure of the valves we included in this study. The Polaris® valve on both radiographs and CT has two discrete metal lobes that indicate the valve setting when compared with index metal markers (Figure 2), making it easy to determine its setting. The CertasTM Plus valve is similar, but the two settings appear very similar, in the same way, that 12:30 and 6:00 look similar on a clock face if the hands of the clock are not visible so that differentiation of the difference in each metal lobe is critical (Figure 4).

We were able to distinguish the difference in the two lobes both in vivo and in vitro, however, using standard CT data. The volume-rendered images of the in vivo scan were very similar in quality to the volume-rendered images from the phantom model, suggesting our phantom adequately provided a simulation of the beam hardening artifacts from the skull and the shunt.

The volume-rendered images generated using images with metal suppression were preferred for the shunt setting evaluation of both the Polaris® and CertasTM Plus valves. iMAR is a metal suppression software product that is offered by Siemens. The iMAR algorithm uses a process of detection and segmentation of the corrupted projection data as a result of the very high attenuation of metal and then modifies the corrupted data by replacing it with calculated estimates of the predicted projection values. The current commercially-available iMAR algorithm [14] uses this image-based metal segmentation method along with multiple iterative processing to improve the quality of the data at each step. The added benefit of a projection-based algorithm is that it can be applied retrospectively, allowing it to be applied to the CT scan data after acquisition.

Dual-energy CT scanners acquire image data at two different X-ray energies (e.g., 80 and 140 kVp) at the same anatomic location simultaneously or nearly simultaneously. With these two datasets, a virtual monoenergetic extrapolation can be performed to create a monochromatic image at much higher virtual energies than currently used for human imaging [15]. Virtual monochromatic images using these calculated high X-rays [16,17] have been demonstrated to reduce the effects of photon starvation and beam hardening but with the trade-off of less tissue contrast [6,18]. For this technique, the decision to use it must be made before scanning. However, we did not have software available to create monoenergetic images, and this remains an avenue for further investigation. We did find that when using data from the higher kVp tube of a dual-energy scanner and processed with metal suppression, those volume-rendered images were selected among the three best for the CertasTM Plus valve.

**Figure 5:**
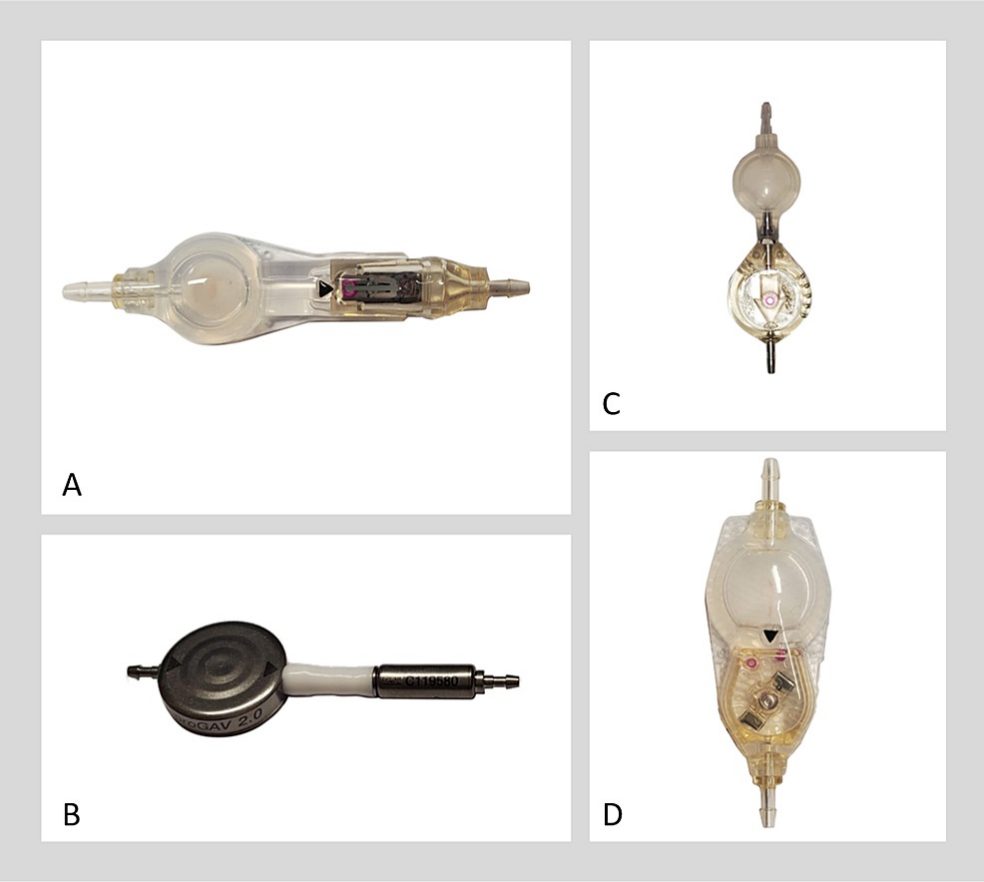
Manufacturer selections for materials and design of their programmable shunts very likely influences the conspicuity of the valve setting on the CT volume rendered images (A) Hakim® (Codman, Raynham, Massachusetts). (B) proGAV 2.0® (Braun, Bethlehem, Pennsylvania). (C) Polaris® (Sophysa, Orsay, France). (D) CertasTM Plus (Codman, Raynham, Massachusetts).

Programmable valves with overlapping metal parts, full metal covers, or those that depend on detection of very fine structures we expect will be difficult to visualize on CT. For example, the proGAV 2.0 ® has a metal covering, and the Hakim® valve parts are too small to appreciate at the resolution of standard CT (Figure 5).

Valves using discrete metal parts with unique arrangements at different settings relative to the indicator markers are more likely to be visible. We found that the CertasTM Plus and Polaris® shunts were most favorable to image with CT among our test valves, most likely because both have non-metallic covers and relatively large and separate metal components. The use of low attenuation metals such as titanium would reduce the effects of photon starvation. However, due to the requirement that the valve’s settings be adjustable with a magnet, ferrous components would seem necessary in all programmable valves. 

This study should not be considered an endorsement of any programmable valve. Many other considerations go into the choice of a valve for a specific patient, and we have not demonstrated at this time that being able to see the valve settings on CT has a substantial benefit in practice. Nevertheless, it does seem to be a favorable feature for a programmable valve design since CT scans are so frequently acquired in patients with hydrocephalus. For example, determination of the valve setting on any head CT scan would allow distinguishing those cases with ventricle enlargement compared with prior CT due to the valve setting adjustment from those cases with shunt malfunction. We believe this potential for imaging should be considered by manufacturers in future design and modification of programmable valves.

There are several limitations to this study. We did not test every programmable shunt on the market. We were only successful in obtaining test valves and setting equipment for the four shunts in use at our institution. This may be due to the costs involved or concern about an unfavorable result, but the design of the valve can be used to predict whether its setting might be visible on CT. But for the intent of this study to demonstrate the feasibility of this approach using CT at standard clinical doses and determine optimal CT technique, we believe the sample was sufficient. 

Second, we did not compare all reconstruction methods for the display of the CT data. As we indicated, we were not able to create monoenergetic images from the dual-energy CT data. We did attempt to use maximum intensity projection instead of volume-rendered reconstructions but found it much more time-consuming to create optimal images and no more effective. Some imagers may find other and better methods to display the data. We believe that for this approach to be effective in practice the ease of image generation and viewing are critical to clinical implementation. We demonstrated in this study that volume-rendered images are readily interpreted by unsophisticated viewers and provided an intuitive correlation with the manufacturer’s published images of valve settings on X-rays.

## Conclusions

In conclusion, we have demonstrated that the valve setting of some programmable shunts can be determined using CT at standard doses in a phantom, interpretation of the VR images can be readily taught to clinical imagers, and this approach was effective in vivo using standard clinical head CT data. 
